# Supplementary material for: Fucoxanthin Ameliorates Atopic Dermatitis Symptoms by Regulating Keratinocytes and Regulatory Innate Lymphoid Cells
Source: Int J Mol Sci. 2020 Mar 22;21(6):2180. doi: 10.3390/ijms21062180 (PMC7139773; doi:10.3390/ijms21062180)
Supplement: Supplementary file 1 [file ijms-21-02180-s001.pdf]

Biochemical Analysis

Serum IgE levels were determined by ELISA using mouse IgE assay kit. (MORINAGA, Yokohama, Japan). After sensitization for 34 days, bromodeoxyuridine was administered intraperitoneally (0.125 g/g weight) to the mice, and auricular lymph node dissection was sampled after 24 h. Samples were digested in 0.1% trypsin/ 0.1% collagenase, and cells were suspended by pipetting. Reaction was stopped by Dulbecco's Modified Eagle's medium (DMEM) (Nacalai Tesque) containing 10% FBS and antibiotics. Measurement of BrdU in suspended cell was determined as directed by the colorimetric cell proliferation ELISA kit, BrdU (Roche Diagnostics Japan, Tokyo, Japan) as described previously [1].

Degranulation Assay

Degranulation of BMMCs was evaluated by measuring the activity of β-hexosaminidase. Generated matured mast cells were cultured in 24-well plates (5×10^5 cells/well) overnight. The cells were sensitized with anti-DNP-IgE (50 ng/mL) (Sigma Chemicals, Saint Louis, MI, USA) for 2 h at 37 °C. After washing the cells with MT buffer (137 mmol/L NaCl, 2.7 mmol/L KCl, 1.8 mmol/L $CaCl_2$, 1 mmol/L $MgCl_2$-$6H_2O$, 5.6 mmol/L glucose, 20 mmol/L HEPES, 0.1% BSA, pH 7.3), they were pretreated with FX for 10 min and then treated with 2.5 μg/mL DNP-labeled human serum albumin (Sigma Chemicals) for 30 min at 37 °C. After incubation, supernatants were transferred to 96-well plates and incubated with 3.3 mM ρ-nitrophenyl-2-acetoamido-2-deoxy-β-d-glucopyranoside for 25 min at 37 °C. The absorbance at 405 nm was measured with a microplate reader. The results were presented as the percentage of total β-hexosaminidase.

Establishment of NF-κB-luc Reporter Cells

RAW264.7 cells were gifted from S. Maeda (Setsunan University, Osaka, Japan). For establishment of NF-κB-luc (Agilent tech., Santa Clara, CA, USA) and stable transfected cells, a neomycin resistant gene cassette from pEGFP-N1 (Takara Clontech Laboratories Inc., Shiga, Japan) was subcloned into the NdeI site by PCR using the following primers: EcoRI-NdeI-neo-F 5′-gcGAATTCATATGgtgtggaaagtccccag-3′, EcoRI-NdeI-BamHI-neo-R 5′-gcGAATTCATATGGATCCtttattctgtct-3′. The gene transfer to RAW264.7 cells was performed by electroporation using Gene Pulser Xcell Electroporation System (Bio-Rad Laboratories Inc., Hercules, CA, USA). Briefly, 200–300 μg NF-κB-luc-Neo^R were transferred to 1-2×10^7 respective cells. Stable transfected cells selected by 400–1000 μg/mL G418 were isolated independently, then generated NF-κB-luc /RAW264.7 cells. Reporter activities were evaluated from optimized time-course analysis after treatment using a One-Glo Luciferase Assay System (Promega, Madison, WI, USA). Luminescence activities were measured using a model GloMax® Discover Microplate Reader (Promega).

Toxicity Assay

HaCaT cells were purchased from Cell Lines Service (Eppelheim, German), and cultured in phenol red free DMEM containing 20% FBS and antibiotics. Cells were plated in 96-well at a density of 10,000 cells, then reacted with FX in the presence of phorbol myristate acetate (PMA) for 23 h. One hour before the end of the reaction, the medium was changed to avoid the absorption of FX, and the cell viability was measured by adding Cell Count Reagent SF (Nacalai Tesque). Supernatants was used to monitor LDH activity by LDH-Glo™ Cytotoxicity Assay (Promega). Cells were then lysed by passive lysis buffer (5% glycerol, 1% Triton X-100, 25 mM Tris-HCl (pH 7.8), 2 mM EDTA, 2 mM DTT). ATP contents in cell lysates were measured with ENLITEN® ATP Assay System (Promega). Absorbance and luminescence were measured using a microplate reader.

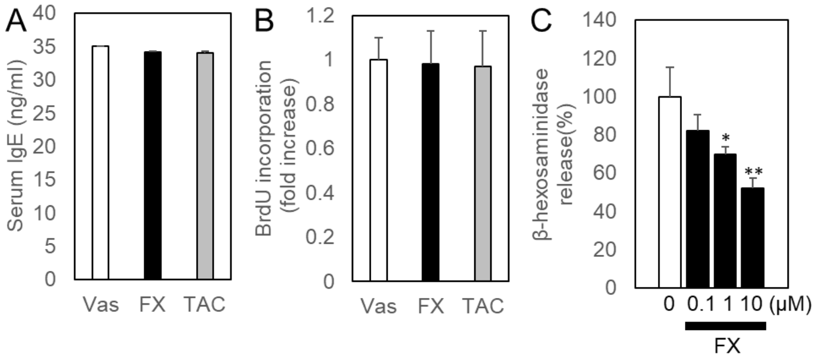

Supplementary Figure 1. Biochemical analysis of serum IgE levels (**A**) and lymphedema (**B**). Each treated mouse showed a similar IgE concentration, and FX failed to attenuate lymph inflammation compared with Vas control. (**C**) FX inhibited BMMCs degranulation. Bone marrow cells were incubated with condition medium containing VEHI-3-CM for four weeks. Degranulation was then triggered by the addition of anti-DNP-IgE. Cells were treated with FX 30 min before Fc receptor stimulation ($n = 6$). Values are means ± SEM. * $p < 0.05$, ** $p < 0.01$ vs DMSO control.

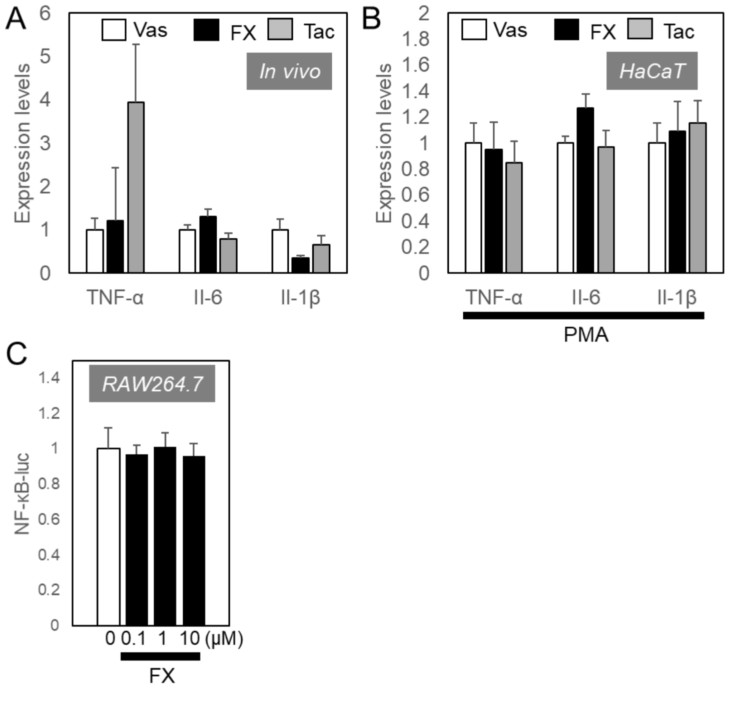

Supplementary Figure 2. The effect of FX or TAC on inflammatory cytokines and NF-κB-luc activities. (**A**) FX or Tac failed to influence TNFα, Il-6, and Il-1β expression levels in Nc/Nga mice. Samples used in Figure 2C were analyzed. (**B**) FX or Tac failed to influence TNFα, IL-6, and IL-1β expression levels in HaCaT cells. Cells were plated in a 6-well plate. After confluent, cells were treated with 1 μM FX or 0.1 μM TAC 30 min before PMA treatment and cultured for 24 h ($n = 6$) (**C**) FX failed to influence NF-κB activities. Using established three NF-κB-luc/RAW264.7 clones, FX actions were analyzed. From preliminary experiments, 0.1 μg/mL lipopolysaccharide (LPS) transiently stimulated NF-κB activities at 8 h exposure, and the effect of FX on LPS-induced activation of NF-κB was examined. Similar results were obtained from all clones ($n = 8$–16). Values are means ± SEM.

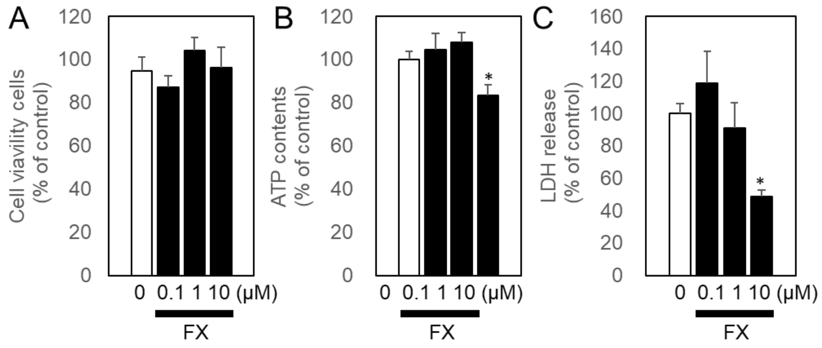

Supplementary Figure 3. Toxicity assays of FX in HaCaT cells. Cells were plated in a 96-well plate at a density of 10,000 cells and treated with indicated concentration of FX 30 min before the addition of 50 ng/mL PMA. Supernatants were stored as the sample for LDH activity assay in deep freezer 23 h later. Cells were then reacted with cell counting reagent SF for 1 h (**A**). Cell lysates were reacted with ATP assay reagent (**B**), and supernatants were reacted with LDH-Glo™ Cytotoxicity Assay reagent (**C**). All procedures were performed according to the manufacturer's instructions. The results show that ATP content was reduced by 10 μM FX probably due to previously reported proton consumption associated with Ucp1 induction [2], but FX was protective against keratinocyte survival as LDH release was suppressed (n = 8). Values are means ± SEM. * $p < 0.05$ vs DMSO control.

Supplementary Table 1. Primers

Mouse

Gene	Forward	Reverse	Accession Number	Product Length (bp)
IFN-γ	CACACCTGATTACTACCTTCT	CCTCAAACTTGGCAATACTC	NM_008337.4	75
Il-1β	CCTCTCCAGCCAAGCTTCCT	TTTGGAAGCAGCCCTTCATC	NM_008361.4	151
Il-2	TTGTGCTCCTTGTCAACAGC	CTGGGGAGTTTCAGGTTCCT	NM_008366.3	175
Il-4	CATCGGCATTTTGAACGAG	CGAGCTCACTCTCTGTGGTG	NM_021283.2	104
Il-5	TTGACAAGCAATGAGACGATGAG	GCCCCTGAAAGATTTCTCCAA	NM_010558.1	82
Il-6	GTGGCTAAGGACCAAGACCA	ACCACAGTGAGGAATGTCCA	NM_001314054.1	135
IL-10	CAGAGCCACATGCTCCTAGA	GTCCAGCTGGTCCTTTGTTT	NM_010548.2	78
Il-13	GTGCCAAGATCTGTGTCTCTCC	TTACAGAGGCCATGCAATATCC	NM_008355.3	215
Il-17	TGAGCTTCCCAGATCACAGA	TCCAGAAGGCCCTCAGACTA	NM_010552.3	101
Il-33	CTGGCCTCACCATAAGAAAGGA	AGGGAGGCAGGAGACTGTGTTAAA	NM_001164724.2	217
TGF-β1	CCCTATATTTGGAGCCTGGA	CTTGCGACCCACGTAGTAGA	NM_011577.2	141
TNF-α	CGGGGTGATCGGTCCCCAAAG	GGAGGGCGTTGGCGCGCTGG	NM_001278601.1	140

TSLP	AGCTTGTCTCCTGAAAATCGAG	AGGTTTGATTCAGGCAGATGTT	NM_021367.2	194
TSLPR	GAGAGCAATGACGATGAGGAC	GAAAGCCTTGTCACCGCTGT	NM_001164735.1	176
FceR1α	TGCCACCGTTCAAGACAG	TTGCGGACATTCCAGTTC	NM_010184.2	180
GATA1	CGCTCCCTGTCACCGGCAGTGC	CCGCCACAGTGGAGTAGCCGTT	NM_008089.2	185
GATA2	CTCCCGACGAGGTGGATGTCTT	CCTGGGCTGTGCAACAAGTGTG	NM_001355253.1	160
HDC	GAGCCCGATGCTAATGAGTC	GAGAAGTTGTCGTCCACAGGTA	NM_008230.6	137
Gapdh	ATGTGTCCGTCGTGGATCTGA	TTGAAGTCGCAGGAGACAACC	NM_001289726.1	145

Human

Gene	Forward	Reverse	Accession Number	Product Length (bp)
IL-1B	CAGCTACGAATCTCCGACCAC	GGCAGGGAACCAGCATCTTC	NM_000576.3	100
IL-6	AACCTGAACCTTCCAAAGATGG	TCTGGCTTGTTCCTCACTACT	NM_000600.5	159
TNFA	ATGAGCACTGAAAGCATGATCC	GAGGGCTGATTAGAGAGAGGTC	NM_000594.4	217
GAPDH	ACCCACTCCTCCACCTTTG	CTCTTGTGCTCTTGCTGGG	NM_001256799.3	178

References

1. Fujita, T.; Meguro, T.; Fukuyama, R.; Nakamuta, H.; Koida, M.; New signaling pathway for parathyroid hormone and cyclic AMP action on extracellular-regulated kinase and cell proliferation in bone cells. Checkpoint of modulation by cyclic AMP. *J. Biol. Chem.* **2002**, *277*, 22191–22200, doi:10.1074/jbc.M110364200.
2. Maeda, H.; Hosokawa, M.; Sashima, T.; Funayama, K.; Miyashita, K. Fucoxanthin from edible seaweed, Undaria pinnatifida, shows antiobesity effect through UCP1 expression in white adipose tissues. *Biochem. Biophys. Res. Commun.* **2005**, *332*, 392–397, doi:10.1016/j.bbrc.2005.05.002.
